# Osteoblast role in the pathogenesis of rheumatoid arthritis

**DOI:** 10.1007/s11033-021-06288-y

**Published:** 2021-03-27

**Authors:** S. Berardi, A. Corrado, N. Maruotti, D. Cici, F. P. Cantatore

**Affiliations:** Rheumatology Clinic - Department of Medical and Surgical Sciences, University of Foggia – Policlinico Riuniti Foggia, Viale Pinto 1, 71121 Foggia, Italy

**Keywords:** Osteoblasts, Rheumatoid arthritis, RANKL/RANK, Bone loss, Cytokines

## Abstract

In the pathogenesis of several rheumatic diseases, such as rheumatoid arthritis, spondyloarthritis, osteoarthritis, osteoporosis, alterations in osteoblast growth, differentiation and activity play a role. In particular, in rheumatoid arthritis bone homeostasis is perturbed: in addition to stimulating the pathologic bone resorption process performed by osteoclasts in course of rheumatoid arthritis, proinflammatory cytokines (such as Tumor Necrosis factor-α, Interleukin-1) can also inhibit osteoblast differentiation and function, resulting in net bone loss. Mouse models of rheumatoid arthritis showed that complete resolution of inflammation (with maximal reduction in the expression of pro-inflammatory factors) is crucial for bone healing, performed by osteoblasts activity. In fact, abnormal activity of factors and systems involved in osteoblast function in these patients has been described. A better understanding of the pathogenic mechanisms involved in osteoblast dysregulation could contribute to explain the generalized and focal articular bone loss found in rheumatoid arthritis. Nevertheless, these aspects have not been frequently and directly evaluated in studies. This review article is focused on analysis of the current knowledge about the role of osteoblast dysregulation occurring in rheumatoid arthritis: a better knowledge of these mechanisms could contribute to the realization of new therapeutic strategies.

## Introduction

Rheumatoid arthritis (RA) is a clinical syndrome including different disease subsets which all lead, by activation of several inflammatory cascades, towards a final common pathway characterized by persistent synovial inflammation and associated damage involving articular cartilage and underlying bone [1].

Progressive joint destruction and general osteoporosis are characteristic signs of RA [2]**;** consequently, it is not surprising that RA patients have a higher risk of vertebral and non-vertebral fractures [3, 4].

The bone remodeling process is guaranteed by bone resorption, in which osteoclasts reabsorb bone, and bone formation, consisting in osteoid production performed by osteoblasts, followed by osteoid mineralization for the replacement of the reabsorbed bone. These two processes are balanced, in order to maintain bone homeostasis; an alteration of this balance occurs in inflammatory rheumatic diseases [5]: in particular, in RA the imbalance leads to net bone loss [6].

The role of factors with proinflammatory action, including Tumor Necrosis Factor-α (TNFα) and Interleukin-1 (IL1), is important for the pathological process of bone resorption carried out by activated osteoclasts in RA [7, 8]. In addition, in course of RA, bone formation process mainly controlled by osteoblasts has not been frequently and directly evaluated in studies and the impact of proinflammatory cytokines on osteoblast function in vivo requires further elucidation [2, 9].

In fact, inflammation not only induces osteoclastogenesis, but also inhibits osteoblast differentiation and function. This inhibition participates to the pathogenesis of arthritic bone loss in RA [5].

Mouse models of RA showed that complete resolution of inflammation (with maximal reduction in the expression of pro-inflammatory factors such as IL1 and TNFα) is crucial for bone healing, performed by osteoblast activity [10].

Osteoblasts could play an important role in the pathogenesis of diseases characterized by bone homeostasis disruption [11]. It was recently proposed that osteoblasts could produce proinflammatory molecules in response to bacterial challenge but also contribute to inflammation through the recruitment of leukocytes to the sites of infection during osteomyelitis, suggesting an active proinflammatory role of osteoblasts [12].

Therefore, a better understanding of the pathogenic mechanisms involved in osteoblast dysregulation could contribute to explain the generalized and focal articular bone loss found in RA patients [13].

This review article is focused on analysis of the current knowledge regarding the role of osteoblast dysregulation occurring in RA, since generalized and focal articular bone loss are typical manifestations of the disease. A better knowledge of these mechanisms could contribute to the realization of new therapeutic strategies.

## Osteoblast role in bone homeostasis

Osteoblasts are cuboid-shaped cells that derive from mesenchymal stem cells (MSC) of the bone marrow, which also form chondrocytes, adipocytes, myocytes. Osteoblast cells are both responsible for bone formation and regulators of osteoclast differentiation and resorption activity. The development of osteoblast cell lineage from MSC is regulated by several factors. For example, specific transcription factors, such as core binding factor α 1 (Cbfa1), Runt related transcription factor 2 (Runx2), Osterix (Osx) are involved. Growth factors, such as Transforming Growth Factor β (TGFβ) and Fibroblast Growth Factor (FGF), specific pathways such as Bone Morphogenetic Protein (BMP) and Wnt system and additional factors like microRNAs (MiRNAs) play a role too [11, 14–16]. Osteoblast cells create and maintain skeletal architecture producing collagenous proteins (mainly type 1 collagen) for bone matrix and regulate the subsequent matrix mineralization, synthetizing non collagenous proteins (including sialoprotein, osteopontin, osteocalcin) associated with the mineralized matrix in vivo [11, 17]. Osteoblasts can differentiate into osteocytes, that are fixed in the bone matrix. Osteocytes play a role in maintaining bone homeostasis, acting as “mechanosensors” connected to osteoblasts and osteoclasts with feedback mechanism [18].

Osteoblast role is crucial not only for bone formation, but also for osteoclast differentiation and activity, by secreting cytokines or by direct cell contact [11].

One of the most important pathways involved in osteoclast differentiation is Receptor activator of nuclear factor kβ ligand (RANKL) /RANK system. RANKL, expressed not only by osteoblast cells (mature and precursors) but also by several cell types (for example osteocytes, synovial fibroblasts, Interleukin-17 produced by activated Th17 cells, B lymphocytes, dendritic cells) [19] binds RANK, expressed by osteoclast precursors, promoting their differentiation into mature osteoclasts. Binding of RANKL to RANK induces receptor oligomerization and activation of TNF receptor associated factor 6 (TRAF6). This factor triggers a series of signaling pathways**:** mitogen activated protein kinases (MAPK) family, nuclear factor kβ (NF-kβ), c-Src in order to promote osteoclastogenesis and bone resorption [20, 21]. MAPK induces the translocation into the nucleus of transcription factors c-fos and c-jun [22]; activated NF-kβ moves into the nucleus, where it upregulates c-fos, that in a complex with Nuclear Factor of Activated T cells 1 (NFATc1) initiates the transcription of a genetic program involved in osteoclastogenesis. Moreover, TRAF6, in complex with c-Src, activates an antiapoptotic program via protein kinase B [20].

Osteoblasts (but also other cells from tissues including hearth, kidney, liver, spleen) are able to express a soluble receptor of RANKL, called osteoprotegerin (OPG), which is one of the most important regulators of RANKL-RANK system [23]. Binding RANKL, OPG avoids RANKL/RANK interaction inhibiting osteoclastogenesis and osteoclast activity [24]. In vitro and in vivo studies suggested that Wnt/β-catenin pathway could regulate OPG expression [25] in osteoblasts, blocking apoptosis and osteoclastogenesis by increasing the OPG/RANKL ratio [26].

Among the various mechanisms that influence cell activity, there is the Wnt system, able to regulate gene expression, cell behaviour, cell adhesion, cell polarity [27], by canonical and non-canonical pathways: the former is mediated by β-catenin, the latter is a term used to include pathways not β-catenin-mediated. Wnt signaling pathway, in particular the canonical one, is involved in osteoblast differentiation from mesenchymal precursors, function and survival; canonical pathway also enhances OPG expression and inhibits osteoclast differentiation [28, 29]. In absence of Wnt stimulation, glycogen synthase kinase 3β (GSK3β) and casein Kinase Iα (CKIα), facilitated by scaffolding proteins axin and tumor suppressor adenomatosis polyposis coli (APC), phosphorylate β-catenin**,** and this whole complex allows the recognition and targeting of phosphorylated β-catenin for ubiquitination and degradation by the proteasome, to prevent cytoplasmic accumulation. In presence of Wnt stimulation, the binding of this ligand to a complex consisting of the seven-pass transmembrane receptor Frizzled (FZD) and single pass transmembrane coreceptor low density lipoprotein receptor related proteins 5 or 6 (LRP 5/6) stimulates FZD that in turn activates the intracellular protein Dishevelled (Dvl) [30]. Dvl, blocking GSK3β, inhibits β-catenin degradation and promotes its cytoplasmic accumulation in the target cells [13, 28, 31]. From cytoplasm, accumulated β-catenin translocates into the nucleus [32], in which β-catenin activates the transcription complex T-cell factor/lymphoid enhanced factor 1 (TCF/Lef1) to initiate transcription and to affect expression of the target genes [33–35].

There are some factors that inhibit Wnt system. Secreted Fz-related protein (sFRP) indirectly prevents the creation of Wnt-FZD complex by binding to Wnt ligands. In addition, proteins of the Dickoppf (DKK) family and Sclerostin (Sost) block canonical Wnt signaling binding directly LRP5/6. DKK-1 itself may stimulate Sost expression to further inhibit Wnt signaling [34, 36, 37].

Osteoblasts are able to express several other factors involved in the process of osteoclastogenesis [38]: macrophage-colony stimulating factor (M-CSF), TNFα, IL1. M-CSF promotes osteoclast precursor proliferation and RANK expression at their surface level. In addition, binding a tyrosin kinase receptor c-fms, M-CSF triggers pathways involved in osteoclast differentiation, ERK1/2 and PI3-K/AKT [11, 39–41].

IL1 also plays a role in TNFα-mediated osteoclastogenesis; in presence of adequate levels of RANKL, IL1 activates p38MAPK (involved in TNFα-mediated osteoclastogenesis) in osteoclast precursors and marrow stromal cells [42].

In vitro and in vivo, TNFα induces osteoclastogenesis and bone resorption [43]. In fact, it enhances RANKL production directly by producing TRAF6, RANK, and NF-κB; these factors activate osteoclast precursor cells in the early phase of osteoclastogenesis; moreover, TNFα indirectly induces RANKL by stimulating osteoclastogenesis-supporting mesenchymal cells [38].

Only TNF receptor type I (TNFRI) is involved in RANKL-induced osteoclastogenesis. It was demonstrated that addition of neutralizing anti-TNFRI antibodies causes suppression of RANKL-induced osteoclastogenesis; the same result is not obtained by using neutralizing anti-TNFRII antibodies [44–46].

Osteoblast OPG/RANKL ratio is also regulated by several hormones: parathormone (PTH), vitamin D, estrogen, calcitonin, serotonin, leptin [11].

BMP pathway (belonging to TGFβ superfamily) is another important mechanism that controls osteoblast growth and differentiation. Binding of BMPs to membrane receptor induces phosphorylation and activation of effectors called SMADs 1, 5 and 8, that recruit and complex with transcriptional cofactor SMAD4; this complex translocates into the nucleus to accomplish gene regulation [47].

BMP ligands such as BMP2, BMP4, BMP6 and BMP7 are pro-osteogenic, whereas BMP3 inhibits BMP pathway [14]. The expression of Runx2 and Osterix is upregulated by BMP pathway to promote osteoblast differentiation [48].

SMAD6 and SMAD7 inhibit receptor complex activation [49]. Moreover, glycoprotein noggin and chordin directly bind BMP ligands to stop BMP pathway [50].

MiRNAs also play a role in osteogenesis, therefore in osteoblast differentiation and activity. MiRNAs are noncoding single-stranded RNA molecules composed of 20–24 nucleotides. They bind to complementary sequences in the 3′ untranslated region (UTR) of mRNAs, negatively affecting gene expression by blocking protein translation and modulating mRNA stability [51]. For example, miR-29a promotes osteoblast proliferation by inhibiting DKK1 expression [52], miR-224 inhibits osteoblast differentiation downregulating SMAD4 [53]. Some miRNAs regulate expression and activity of several factors, such as Runx2, BMP2, RANKL, therefore affecting osteoclast and osteoblast differentiation and function [54].

## Bone loss in RA

In RA, from the early stages of disease, the alteration of bone homeostasis, caused by an imbalance of the processes of bone resorption and bone formation in favor of the first, is responsible for a net bone loss [6, 18].

In fact, inflamed synovial tissues produce proinflammatory factors (mainly TNFα, IL1, IL6) able to interfere with osteoblast and osteoclast differentiation and function.

In the “pre-clinical” phase of RA, the loss of tolerance produces typical autoantibodies, including rheumatoid factor (RF) and anti-citrullinated protein antibodies (ACPAs), that also play a role in RA-mediated bone loss [14, 18]. The presence of both antibodies seems to be associated with high erosive disease burden but the role of RF on bone erosions is not clear (for example, if it could have an additive effect in ACPA positive subjects or not) [55, 56].

In ACPA positive RA patient, also before clinical onset of disease, bone architecture is altered, producing bone loss [57]. ACPAs are predictor of a more aggressive form of disease, in particular about the development of bone erosion [58]. In fact, it was shown that ACPAs both enhance TNFα production by macrophages (promoting osteoclast differentiation) [59] and directly interact with citrullinated proteins on osteoclast precursors membrane, enhancing osteoclast differentiation [18].

Altered bone homeostasis is expressed through three main alterations of bone remodeling, which can also be appreciated in X-ray images: focal bone erosions at the joint margin, where inflamed synovium takes direct contact with bone; periarticular bone loss at the level of the affected joints, probably caused by proinflammatory factors expressed by inflamed joints; generalized bone loss, likely due to the passage of these factors in the systemic circulation, affecting bone metabolism at distal sites [5, 60].

## Osteoblast role in RA

Osteoblasts play a key role in the pathogenesis of focal articular bone loss, in association to osteoclasts, macrophages, synovial cells [13].

It has been shown that healing of focal bone erosions is possible, but only when the inflammation is well controlled; indeed, in patients in whom repair does not occur, osteoblast function may be inhibited by persisting subclinical inflammation in the involved joints [9, 61]. Using murine model of RA and dynamic bone histomorphometry, Walsh et al. showed that bone formation rate, evaluated at bone surfaces adjacent to inflammation, is not different to those observed in non-arthritic bone, suggesting that increased osteoclast resorption at these sites is not well compensated by the action of osteoblasts. Interestingly, the Authors also reported the negative effect of inflammation status on osteoblast function as, within arthritic bone, there was a reduction in bone formation rate in areas adjacent to inflammation places, whereas it was regular in bone areas near the normal bone marrow. In areas characterized by bone erosions they detected a considerable amount of osteoblastic precursor cells but few mature osteoblast cells [6], while the number of mature osteoblastic cells, ready to repair erosive damage, was increased when they assessed the same areas of bone erosion when the inflammation was extinguished [9].

**Fig. 1:**
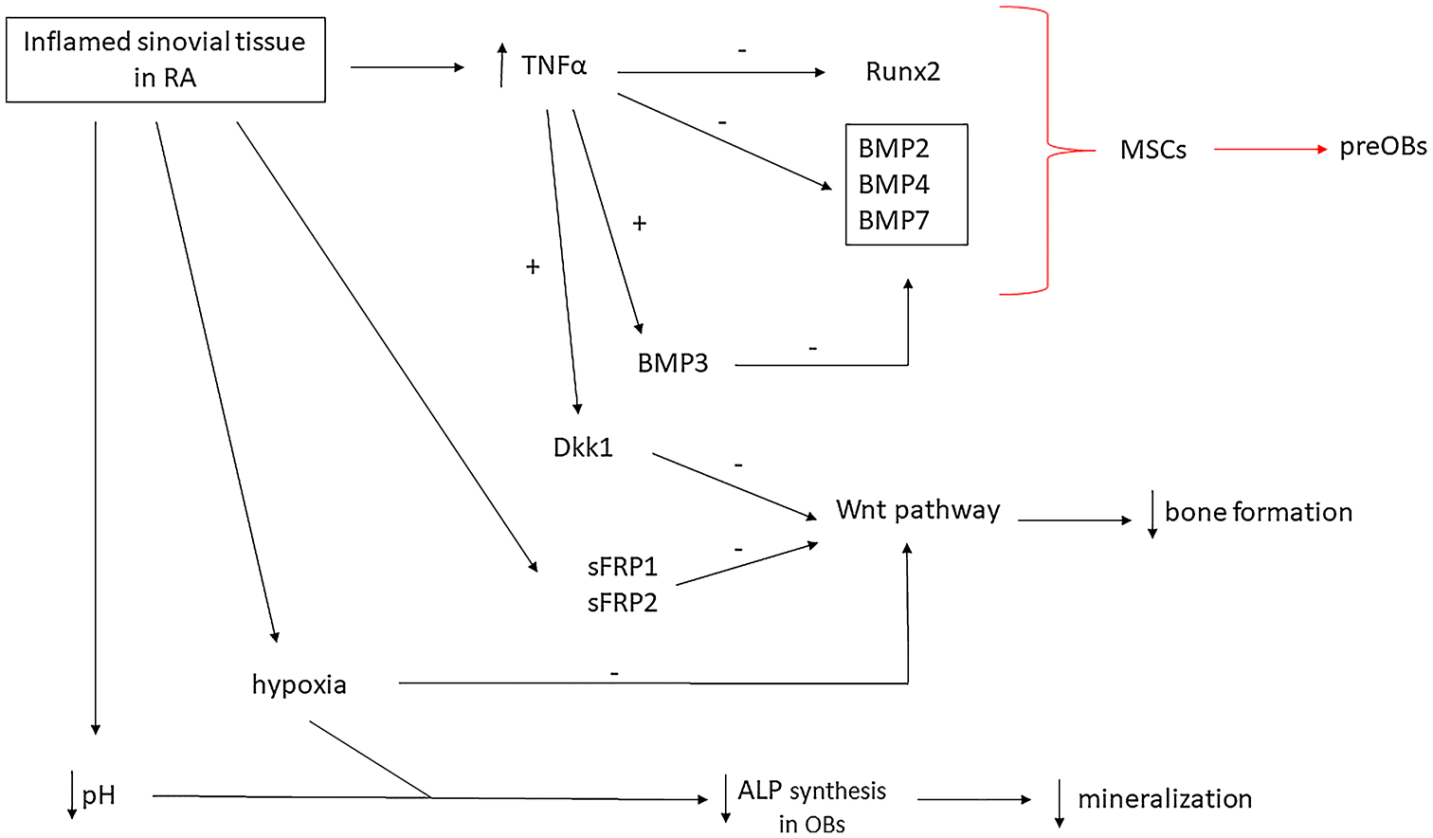
Inflamed sinovial tissue in RA (Rheumatoid Arthritis) leads to enhanced expression of TNFα (Tumor necrosis factor α) which inhibits Runx2 (Runt related transcription factor 2) and BMPs (Bone Morphogenetic Protein), therefore reducing MSC (mesenchymal stem cells) differentiation into preOBs (preosteoblasts). Moreover, TNFα enhances BMP3 mRNA expression in mature osteoblasts, further inhibiting BMP pathway. Sinovitis also induces the Wnt inhibitors sFRP (secreted Fz-related protein) 1 and 2, therefore reducing bone formation. The Wnt pathway is furthermore inhibited by Dkk1 (Dickoppf1) TNFα-induced expression and by inflammation-related hypoxia. Hypoxia and acidosis secondary to joint flogosis cause reduced ALP (alkaline phosphatase) synthesis in OBs, consequently contrasting bone mineralization. Red arrows: inhibited processes; ↑: increased; ↓: reduced

Therefore, in RA patients, cells involved in the inflammatory process produce factors inhibiting osteoblast differentiation and function at the sites of focal bone erosions (Fig. 1), such as Wnt signaling pathway antagonists sFRP1 and sFRP2. These factors are secreted by inflamed synovial tissues [11] and their expression is downregulated when inflammation is completely resolved, suggesting the importance of inflammation resolution to enhance erosion repair. In this situation, expression of Wnt antagonist Wnt10b, conversely, is upregulated [5].

In this regard, the formation of a bone erosion microenvironment was shown when osteoblasts were co-coltured with RA derived synovial tissue, which produces molecules such as inflammatory factors (TNFα, IL1), exosomes and MiRNAs. These factors could inhibit osteoblast cell proliferation by regulating MAPKs pathway, in particular inhibiting MAPK Jnk and p-38. This is one of the processes that could explain the phenomenon of bone erosion occurring in RA [62].

The inhibition of osteoblast cells differentiation and activity by cytokines such as TNFα and IL1 is confirmed by in vitro studies [5]. In pre-osteoblast cultures including TNFα, osteoblast differentiation and maturation were arrested [63] and osteoblast maturation markers (collagen type I, alkaline phosphatase and osteocalcin) were decreased [64]. TNFα enhances degradation of Runx2, a transcription factor which plays a role in osteoblast differentiation [65]. Moreover, TNFα induces Wnt system antagonist DKK1 in osteoblasts, leading to reduction of osteoblast-mediated bone formation. DKK1 is significantly detectable in serum and inflamed synovium of RA patients overexpressing TNFα compared to healthy subjects and blocking DKK1 with specific antibodies protects from local bone resorption (reducing osteoclast numbers in the joints) and from negative DKK1 effects on osteoblastogenesis and osteoblast function, promoting bone repair [66]. Exposition of osteoblast-like cells to sera of RA patients in therapy with TNFα inhibitor was related to a decreased synthesis of the cytokine IL6, responsible for bone loss process in arthritis [67]. IL6, reducing OPG/RANKL ratio, promotes osteoblast-mediated osteoclasts differentiation, enhancing bone loss [68]. Moreover, IL6 seems to play a role in the inhibition of osteoblast differentiation by TNFα; Malysheva et al. showed, through in vitro experiment, that knockdown of IL6 expression partially protects osteoblast differentiation from the negative effect of TNFα. IL6 together with TNFα and DKK1 inhibits osteogenic Wnt signaling, expressing its anti-osteoblastic effects in course of RA [69].

Osteoblast differentiation and function is also dependent on the BMP signaling pathway. BMP2, BMP4, BMP7 promote osteoblast differentiation from mesenchymal precursor cells, while BMP3 inhibits this pathway. BMP3 could have a role in inflammatory arthritis, particularly in the inhibition of osteoblasts function, which leads to a difficult healing of bone erosions in RA. In fact, proinflammatory cytokine TNFα (Fig. 1), in course of RA, in addition to inhibiting BMP-induced bone formation [70], induces BMP3 expression in mature osteoblast at the sites of bone erosion, as showed by Matzelle et al., suggesting a crucial role of BMP3 in bone repair failure in RA patients [71]. In addition, as suggested by Katsuyama et al. [72], Fibroblast Growth Factor 8 (FGF8) could participate to inhibit BMP-induced osteoblast differentiation (in particular inhibiting BMP2) by interacting with TNFα receptor signaling. FGF8 belongs to a family involved in several biological processes and plays a role in bone and cartilage homeostasis [73]. A possible effect of FGF8 in osteoblast proliferation and differentiation was described [74] and an involvement of FGF8 together with BMPs and inflammatory cytokines in both RA and osteoarthritis (OA) was suggested [75, 76].

Other factors able to affect osteoblast activity are pH and oxygen: in arthritic bone, the microenvironment is characterized by reduction of pH and hypoxia. It was shown that, in vitro experiments, low pH and hypoxia decrease alkaline phosphatase (ALP) synthesis in osteoblasts, inhibiting mineralization [77, 78]. Hypoxia blocks Wnt pathway in osteoblast-like cells, both upregulating DKK1 [79] and inhibiting transcriptional activity by blocking β-catenin [80].

On the other hand, contradictory in vitro findings suggested that TNFα can also activate osteoblastogenesis if present in low concentration or utilized in short treatment, enhancing expression of ALP, Runx2 and osteocalcin [81].

Many other factors and mechanisms are involved in the imbalance of bone homeostasis occurring in RA, therefore affecting osteoblasts development and activity, but less data are available: these are aspects to further investigate, also for their possible therapeutic usefulness in control of bone damage in RA and other arthritis.

Possible therapeutic agents for this purpose, particularly to prevent inflammation-mediated bone loss, could be NOTCH inhibitors and Nrf2 activators. NOTCH-dependent signaling pathways are involved in bone cells function; studies on mice demonstrated that activation of NOTCH signaling decreased osteoblast numbers, conversely loss of NOTCH signaling in MSCs or osteoblast precursors led to an increase in bone mass. Zhang et al. showed that in MSCs from RA mice, expression of genes encoding NOTCH pathway members was markedly elevated and persistent NOTCH activation in MSCs contributed to inhibit osteoblast differentiation associated with RA [82].

Nrf2 is a transcription factor with anti-oxidative function [83]. The activation of Nrf2 in osteoclasts inhibits osteoclastogenesis and bone destruction suppressing oxidase stress (ROS) signaling, therefore inhibiting RANKL–dependent osteoclastogenesis [84]. Narimiya et al. evaluated if the activation of Nrf2 in osteoblasts reduces inflammatory cytokine production, in particular the expression of IL6, which promotes osteoclastogenesis, demonstrating that Nrf2 activation has a direct and indirect effect on osteoclastogenesis, involving both osteoclasts and osteoblasts. Therefore, a Nrf2 activator could have a therapeutic effect against inflammatory bone loss, such as in RA patients [85].

Immune cells are key actors for the development and maintenance of the inflammatory process in RA, so it should not be surprising the possible involvement of B cells and mononuclear cells also in the mechanisms that lead to imbalance of bone homeostasis in course of inflammatory arthritis.

Given that B cells play several roles in RA, such as promotion of osteoclastogenesis through expression of TNFα and RANKL, their effect on osteoblast differentiation and function is still unclear. Experiments by Sun et al. regarding two murine RA models (collagen-induced arthritis – CIA—and the TNF-transgenic mice) showed that in RA patients B cells are able to produce osteoblast inhibitors, suppressing bone formation. In fact, in both RA models, they demonstrated an accumulation of B cells in subchondral and endosteal bone marrow area, especially close to bone surface and adjacent to “osteocalcin + ” osteoblasts. Osteoblast inhibitors such as chemokine ligand 3 (CCL3) and TNFα from subchondral areas were expressed by RA B cells. These factors inhibited osteoblast differentiation from MSCs through activation of NF-κB and ERK signaling pathways; CCL3 and TNFα deletion improved this effect in vivo. Furthermore, in RA mice, B cell depletion reduces bone erosion and osteoblast inhibition [86, 87], confirming its role in bone loss.

Migration of mononuclear cells plays an important step in inflammation in course of RA [88] and some studies suggest acting on this mechanism to control bone loss. Monocyte chemoattractant protein-1 (MCP-1), also known as chemokine ligand 2 (CCL2), is one of the factors involved in monocytes chemotaxis [89] and increased levels of it were found in synovial tissue and fluid of RA patients compared to healthy controls [90, 91]. The extracellular matrix component Cysteine-rich protein 61 (Cyr61/CCN1), involved in endothelial cell function [92], seems to be also involved in RA pathogenesis, regulating proinflammatory cytokines effect. Chen et al. showed that CCN1 induces upregulation of CCL2 expression in osteoblasts, through the negative regulation of miR-518a-5p via the MAPK signaling pathway, enhancing monocyte migration. Moreover, they showed that recombinant lentiviral knockdown of CCN1 reduced bone erosion and monocyte infiltration in joints of mice with CIA, suggesting CCN1 as a target for RA treatment [93].

Besides the well-known actions of IL1 and IL6 in inflammatory process and related bone homeostasis disfunction, other components of IL family may play a role, affecting not only osteoclast but also osteoblast differentiation and activity. IL35 is an antinflammatory cytokine that inhibits TNFα-induced osteoclastogenesis [94]; in addition, Li et al. showed that IL35, through Wnt/β-catenin signaling pathway, could stimulate basal and TNFα-activated osteoblast differentiation at early stage and significantly stimulated matrix mineralization, enhancing osteoblast differentiation at a late stage too. In this way, IL35 could mitigate RA bone loss progression [81]. IL23 is a cytokine that plays a pro-osteoclastogenic role in course of inflammation, increasing joint damage by osteoclasts [95]. Although no direct effects of IL23 on osteoblasts have been documented [96, 97], this cytokine can exert indirect effects through downstream cytokines such as IL17. There are six isoforms of IL17 (IL17-A, IL17-B, IL17-C, IL17-D, IL17-E, IL17-F), and IL17-A is the isoform best studied. IL17, in physiological conditions, is involved in defending epithelia and mucous membranes against bacteria and fungi, but it is overexpressed and seems to play an important role in inflammatory diseases like RA and spondyloarthritis (SpA); it was suggested that IL17-A is involved in the alteration of bone homeostasis in inflammatory arthritis by acting on osteoclast and osteoblast related pathways. In vitro stimulation of osteoblasts with IL17-A enhances sFRP1, a Wnt pathway antagonist, inhibiting osteoblasts formation [98]. Actually, the effects of IL17-A on osteoblasts in in vitro models are not always homogeneous [99], and the role of IL17 family in bone remodeling is still not clear and needs for further investigations, but its overexpression in pathologic conditions is harmful to bone homeostasis.

## Future perspective

There is accumulating evidence on the role of osteoblasts in RA, SpA, OA and osteoporosis [13]. In fact, osteoblast differentiation, growth and activity are often dysregulated in these diseases. In RA, the balance between bone resorption and bone formation is perturbed, leading to bone loss.

Beside induction of osteoclastogenesis, inflammation inhibits osteoblast differentiation and function, and this can lead to arthritic bone loss in RA. In fact, cells involved in the inflammatory process produce factors inhibiting osteoblast development and activities. In mouse models of RA, only the complete resolution of inflammation allows bone healing, performed by osteoblasts, whereas persisting subclinical inflammation inhibits osteoblast differentiation and function. However, there is a need to expand research on this topic, as most studies focus more on osteoclast than osteoblast dysregulation in course of RA. Several factors and signal pathways, previously unknown or considered irrelevant (for example the members of IL17 family), are believed to be involved in the mechanisms that regulate the action of bone cells in inflammatory arthritis and that lead to net bone loss in the case of RA. Even if osteoclasts are the cells responsible for bone resorption (for this reason in the past the attention has been focused in particular on these cells), bone tissue is extremely dynamic and interconnected, so the role of osteoblasts is also crucial. All factors involved in their maturation and function, and the role of these cells in the pathogenesis of RA should be clarified as much as possible, in order to find further new therapeutic possibilities to fight not only RA but also several other inflammatory diseases.

## References

[1] Scott DL, Wolfe F, Huizinga TWJ (2010). Rheumatoid arthritis. Lancet.

[2] Morimoto D, Kuroda S, Kizawa T (2009). Equivalent osteoblastic differentiation function of human mesenchymal stem cells from rheumatoid arthritis in comparison with osteoarthritis. Rheumatology.

[3] Hoes JN, Bultink IEM, Lems WF (2015). Management of osteoporosis in rheumatoid arthritis patients. Expert Opin Pharmacother.

[4] Chen B, Cheng G, Wang H, Feng Y (2016). Increased risk of vertebral fracture in patients with rheumatoid arthritis: a meta-analysis. Medicine (Baltimore).

[5] Baum R, Gravallese EM (2016). Bone as a target organ in rheumatic disease: impact on osteoclasts and osteoblasts. Clin Rev Allergy Immunol.

[6] Walsh NC, Reinwald S, Manning CA (2009). Osteoblast function is compromised at sites of focal bone erosion in inflammatory arthritis. J Bone Miner Res.

[7] Zhao B (2017). TNF and bone remodeling. Curr Osteoporos Rep.

[8] O’Gradaigh D, Ireland D, Bord S, Compston JE (2004). Joint erosion in rheumatoid arthritis: Interactions between tumour necrosis factor α, interleukin 1, and receptor activator of nuclear factor κB ligand (RANKL) regulate osteoclasts. Ann Rheum Dis.

[9] Matzelle MM, Gallant MA, Condon KW (2012). Resolution of inflammation induces osteoblast function and regulates the Wnt signaling pathway. Arthritis Rheum.

[10] Turner JD, Naylor AJ, Christopher B, Filer A, Tak PP (2018). Fibroblasts and osteoblasts in inflammation and bone damage. Stromal Immunol.

[11] Neve A, Corrado A, Cantatore FP (2011). Osteoblast physiology in normal and pathological conditions. Cell Tissue Res.

[12] He X, Liu J, Liang C (2019). Osteoblastic PLEKHO1 contributes to joint inflammation in rheumatoid arthritis. EBioMedicine.

[13] Corrado A, Maruotti N, Cantatore FP (2017). Osteoblast role in rheumatic diseases. Int J Mol Sci.

[14] Shaw AT, Gravallese EM (2016). Mediators of inflammation and bone remodeling in rheumatic disease. Semin Cell Dev Biol.

[15] Wrana JL (1988). Differential effects of transforming growth factor-13 on the synthesis of extracellular matin proteins by normal fetal rat calvarial bone cell populations. J Cell Biol.

[16] Globus RK, Patterson-buckendahl P, Gospodarowicz D (1988). Regulation of bovine bone cell proliferation by fibroblast growth factor and transforming growth. Endocrinology.

[17] Capulli M, Paone R, Rucci N (2014). Osteoblast and osteocyte: games without frontiers. Arch Biochem Biophys.

[18] Shim JH, Stavre Z, Gravallese EM (2018). Bone loss in rheumatoid arthritis: basic mechanisms and clinical implications. Calcif Tissue Int.

[19] Geusens P (2012). The role of RANK ligand/osteoprotegerin in rheumatoid arthritis. Ther Adv Musculoskelet Dis.

[20] Nagy V, Penninger M (2015). The RANKL-RANK story. Gerontology.

[21] Yavropoulou MP, Yovos JG (2008). Osteoclastogenesis: current knowledge and future perspectives. J Musculoskelet Neuronal Interact.

[22] Matsumoto M, Sudo T, Maruyama M (2000). Activation of p38 mitogen-activated protein kinase is crucial in osteoclastogenesis induced by tumor necrosis factor. FEBS Lett.

[23] Wada T, Nakashima T, Hiroshi N, Penninger JM (2006). RANKL – RANK signaling in osteoclastogenesis and bone disease. Trends Mol Med.

[24] Khosla S (2014). Minireview: the OPG/RANKL/RANK system. Endocrinology.

[25] Glass DA, Bialek P, Ahn JD (2005). Canonical Wnt signaling in differentiated osteoblasts controls osteoclast differentiation. Dev Cell.

[26] Kubota T, Michigami T, Ozono K (2009). Wnt signaling in bone metabolism. J Bone Miner Metab.

[27] Moon RT, Bowerman B, Boutros M, Perrimon N (2002). The promise and perils of Wnt signaling through beta-catenin. Science.

[28] Maeda K, Kobayashi Y, Koide M (2019). The regulation of bone metabolism and disorders by Wnt signaling. Int J Mol Sci.

[29] Westendorf JJ, Kahler RA, Schroeder TM (2004). Wnt signaling in osteoblasts and bone diseases. Gene.

[30] Tamai K, Semenov M, Kato Y, Spokony R, Liu C, Katsuyama Y, Hess F, Saint-Jeannet JP, He X (2000). LDL-receptor-related proteins in Wnt signal transduction. Nature.

[31] Logan CY, Nusse R (2004). The Wnt signaling pathway in development and disease. Annu Rev Cell Dev Biol.

[32] Miao CG, Yang YY, He X, Li XF, Huang C, Huang Y, Zhang L, Lv XW, Jin Y, Li J (2013). Wnt signaling pathway in rheumatoid arthritis, with special emphasis on the different roles in synovial inflammation and bone remodeling. Cell Signal.

[33] Maeda K, Takahashi N, Kobayashi Y (2013). Roles of Wnt signals in bone resorption during physiological and pathological states. J Mol Med (Berl).

[34] Karner CM, Long F (2017). Wnt signaling and cellular metabolism in osteoblasts. Cell Mol Life Sci.

[35] Pai SG, Carneiro BA, Mota JM, Costa R, Leite CA, Barroso-Sousa R, Kaplan JB, Chae YK, Giles FJ (2017). Wnt/beta-catenin pathway: modulating anticancer immune response. J Hematol Oncol.

[36] Johnson ML, Harnish K, Nusse R, Van Hul W (2004). LRP5 and Wnt signaling: a union made for bone. J Bone Miner Res.

[37] Heiland GR, Zwerina K, Baum W, Kireva T, Distler JH, Grisanti M, Asuncion F, Li X, Ominsky M, Richards W, Schett G, Zwerina J (2010). Neutralisation of Dkk-1 protects from systemic bone loss during inflammation and reduces sclerostin expression. Ann Rheum Dis.

[38] Maruotti N, Corrado A, Neve A, Cantatore FP (2012). Bisphosphonates: effects on osteoblast. Eur J Clin Pharmacol.

[39] Tsurukai T, Udagawa N, Matsuzaki K, Takahashi N, Suda T (2000). Roles of macrophage-colony stimulating factor and osteoclast differentiation factor in osteoclastogenesis. J Bone Miner Metab.

[40] Faccio R, Takeshita S, Zallone A, Ross FP, Teitelbaum SL (2003). c-Fms and the alphavbeta3 integrin collaborate during osteoclast differentiation. J Clin Invest.

[41] Boyle WJ, Simonet WS, Lacey DL (2003). Osteoclast differentiation and activation. Nature.

[42] Wei S, Kitaura H, Zhou P, Ross FP, Teitelbaum SL (2005). IL-1 mediates TNF-induced osteoclastogenesis. J Clin Invest.

[43] Cao Y, Jansen IDC, Sprangers S, de Vries TJ, Everts V (2017). TNF-α has both stimulatory and inhibitory effects on mouse monocyte-derived osteoclastogenesis. J Cell Physiol.

[44] Zou W, Hakim I, Tschoep K, Endres S, Bar-Shavit Z (2001). Tumor necrosis factor-alpha mediates RANK ligand stimulation of osteoclast differentiation by an autocrine mechanism. J Cell Biochem.

[45] Nakao A, Fukushima H, Kajiya H, Ozeki S, Okabe K (2007). RANKL-stimulated TNFalpha production in osteoclast precursor cells promotes osteoclastogenesis by modulating RANK signaling pathways. Biochem Biophys Res Commun.

[46] Asagiri M, Takayanagi H (2007). The molecular understanding of osteoclast differentiation. Bone.

[47] Lowery JW, Rosen V (2018). The BMP pathway and its inhibitors in the skeleton. Physiol Rev.

[48] Matsubara T, Kida K, Yamaguchi A, Hata K, Ichida F, Meguro H, Aburatani H, Nishimura R, Yoneda T (2008). BMP2 regulates Osterix through Msx2 and Runx2 during osteoblast differentiation. J Biol Chem.

[49] Miyazawa K, Miyazono K (2017). Regulation of TGF-β family signaling by inhibitory smads. Cold Spring Harb Perspect Biol.

[50] Gazzerro E, Gangji V, Canalis E (1998). Bone morphogenetic proteins induce the expression of noggin, which limits their activity in cultured rat osteoblasts. J Clin Invest.

[51] Lian JB, Stein GS, van Wijnen AJ, Stein JL, Hassan MQ, Gaur T, Zhang Y (2012). MicroRNA control of bone formation and homeostasis. Nat Rev Endocrinol.

[52] Zhang F, Cao K, Du G, Zhang Q, Yin Z (2019). miR-29a promotes osteoblast proliferation by downregulating DKK-1 expression and activating Wnt/β-catenin signaling pathway. Adv Clin Exp Med.

[53] Luo Y, Cao X, Chen J, Gu J, Zhao J, Sun J (2018). MicroRNA-224 suppresses osteoblast differentiation by inhibiting SMAD4. J Cell Physiol.

[54] Zhao H, Lu A, He X (2020). Roles of microRNAs in bone destruction of rheumatoid arthritris. Front Cell Dev Biol.

[55] Hecht C, Englbrecht M, Rech J, Schmidt S, Araujo E, Engelke K, Finzel S, Schett G (2015). Additive effect of anti-citrullinated protein antibodies and rheumatoid factor on bone erosions in patients with RA. Ann Rheum Dis.

[56] van Steenbergen HW, Ajeganova S, Forslind K, Svensson B, van der Helm-van Mil AH (2015). The effects of rheumatoid factor and anticitrullinated peptide antibodies on bone erosions in rheumatoid arthritis. Ann Rheum Dis.

[57] Kleyer A, Finzel S, Rech J, Manger B, Krieter M, Faustini F, Araujo E, Hueber AJ, Harre U, Engelke K, Schett G (2014). Bone loss before the clinical onset of rheumatoid arthritis in subjects with anticitrullinated protein antibodies. Ann Rheum Dis.

[58] Jilani AA, Mackworth-Young CG (2015). The role of citrullinated protein antibodies in predicting erosive disease in rheumatoid arthritis: a systematic literature review and meta-analysis. Int J Rheumatol.

[59] Harre U, Georgess D, Bang H (2012). Induction of osteoclastogenesis and bone loss by human autoantibodies against citrullinated vimentin. J Clin Invest.

[60] Panagopoulos PK, Lambrou GI (2018). Bone erosions in rheumatoid arthritis: recent developments in pathogenesis and therapeutic implications. J Musculoskelet Neuronal Interact.

[61] Ideguchi H, Ohno S, Hattori H, Senuma A, Ishigatsubo Y (2006). Bone erosions in rheumatoid arthritis can be repaired through reduction in disease activity with conventional disease-modifying antirheumatic drugs. Arthritis Res Ther.

[62] Zheng W, Gu X, Hu D, Hao Y (2019). Co-culture with synovial tissue in patients with rheumatoid arthritis suppress cell proliferation by regulating MAPK pathway in osteoblasts. Am J Transl Res.

[63] Gilbert L, He X, Farmer P, Boden S, Kozlowski M, Rubin J, Nanes MS (2000). Inhibition of osteoblast differentiation by tumor necrosis factor-alpha. Endocrinology.

[64] Wehmeyer C, Pap T, Buckley CD, Naylor AJ (2017). The role of stromal cells in inflammatory bone loss. Clin Exp Immunol.

[65] Gilbert L, He X, Farmer P, Rubin J, Drissi H, van Wijnen AJ, Lian JB, Stein GS, Nanes MS (2002). Expression of the osteoblast differentiation factor RUNX2 (Cbfa1/AML3/Pebp2alpha A) is inhibited by tumor necrosis factor-alpha. J Biol Chem.

[66] Diarra D, Stolina M, Polzer K, Zwerina J, Ominsky MS, Dwyer D, Korb A, Smolen J, Hoffmann M, Scheinecker C, van der Heide D, Landewe R, Lacey D, Richards WG, Schett G (2007). Dickkopf-1 is a master regulator of joint remodeling. Nat Med.

[67] Musacchio E, Valvason C, Botsios C, Ostuni F, Furlan A, Ramonda R, Modesti V, Sartori L, Punzi L (2009). The tumor necrosis factor-{alpha}-blocking agent infliximab inhibits interleukin 1beta (IL-1beta) and IL-6 gene expression in human osteoblastic cells. J Rheumatol.

[68] Liu XH, Kirschenbaum A, Yao S, Levine AC (2005). Cross-talk between the interleukin-6 and prostaglandin E(2) signaling systems results in enhancement of osteoclastogenesis through effects on the osteoprotegerin/receptor activator of nuclear factor-{kappa}B (RANK) ligand/RANK system. Endocrinology.

[69] Malysheva K, de Rooij K, Lowik CW (2016). Interleukin 6/Wnt interactions in rheumatoid arthritis: interleukin 6 inhibits Wnt signaling in synovial fibroblasts and osteoblasts. Croat Med J.

[70] Nakase T, Takaoka K, Masuhara K, Shimizu K, Yoshikawa H, Ochi T (1997). Interleukin-1 beta enhances and tumor necrosis factor-alpha inhibits bone morphogenetic protein-2-induced alkaline phosphatase activity in MC3T3-E1 osteoblastic cells. Bone.

[71] Matzelle MM, Shaw AT, Baum R (2016). Inflammation in arthritis induces expression of BMP3, an inhibitor of bone formation. Scand J Rheumatol.

[72] Katsuyama T, Otsuka F, Terasaka T, Inagaki K, Takano-Narazaki M, Matsumoto Y, Sada KE, Makino H (2015). Regulatory effects of fibroblast growth factor-8 and tumor necrosis factor-α on osteoblast marker expression induced by bone morphogenetic protein-2. Peptides.

[73] Ellman MB, Yan D, Ahmadinia K, Chen D, An HS, Im HJ (2013). Fibroblast growth factor control of cartilage homeostasis. J Cell Biochem.

[74] Valta MP, Hentunen T, Qu Q, Valve EM, Harjula A, Seppänen JA, Väänänen HK, Härkönen PL (2006). Regulation of osteoblast differentiation: a novel function for fibroblast growth factor 8. Endocrinology.

[75] Verschueren PC, Lories RJ, Daans M, Théate I, Durez P, Westhovens R, Luyten FP (2009). Detection, identification and in vivo treatment responsiveness of bone morphogenetic protein (BMP)-activated cell populations in the synovium of patients with rheumatoid arthritis. Ann Rheum Dis.

[76] Schmal H, Pilz IH, Mehlhorn AT, Dovi-Akue D, Kirchhoff C, Südkamp NP, Gerlach U, Niemeyer P (2012). Expression of BMP-receptor type 1A correlates with progress of osteoarthritis in human knee joints with focal cartilage lesions. Cytotherapy.

[77] Utting JC, Robins SP, Brandao-Burch A, Orriss IR, Behar J, Arnett TR (2006). Hypoxia inhibits the growth, differentiation and bone-forming capacity of rat osteoblasts. Exp Cell Res.

[78] Brandao-Burch A, Utting JC, Orriss IR, Arnett TR (2005). Acidosis inhibits bone formation by osteoblasts in vitro by preventing mineralization. Calcif Tissue Int.

[79] Colla S, Zhan F, Xiong W, Wu X, Xu H, Stephens O, Yaccoby S, Epstein J, Barlogie B, Shaughnessy JD (2007). The oxidative stress response regulates DKK1 expression through the JNK signaling cascade in multiple myeloma plasma cells. Blood.

[80] Almeida M, Han L, Martin-Millan M, O'Brien CA, Manolagas SC (2007). Oxidative stress antagonizes Wnt signaling in osteoblast precursors by diverting beta-catenin from T cell factor- to forkhead box O-mediated transcription. J Biol Chem.

[81] Li Y, Yuan L, Jiang S, Liu S, Xia L, Shen H, Lu J (2019). Interleukin-35 stimulates tumor necrosis factor-α activated osteoblasts differentiation through Wnt/β-catenin signaling pathway in rheumatoid arthritis. Int Immunopharmacol.

[82] Zhang H, Hilton MJ, Anolik JH (2014). NOTCH inhibits osteoblast formation in inflammatory arthritis via noncanonical NF-κB. J Clin Invest.

[83] Kobayashi EH, Suzuki T, Funayama R, Nagashima T, Hayashi M, Sekine H, Tanaka N, Moriguchi T, Motohashi H, Nakayama K, Yamamoto M (2016). Nrf2 suppresses macrophage inflammatory response by blocking proinflammatory cytokine transcription. Nat Commun.

[84] Kanzaki H, Shinohara F, Kajiya M, Kodama T (2013). The Keap1/Nrf2 protein axis plays a role in osteoclast differentiation by regulating intracellular reactive oxygen species signaling. J Biol Chem.

[85] Narimiya T, Kanzaki H, Yamaguchi Y (2019). Nrf2 activation in osteoblasts suppresses osteoclastogenesis via inhibiting IL-6 expression. Bone Rep.

[86] Marston B, Palanichamy A, Anolik JH (2010). B cells in the pathogenesis and treatment of rheumatoid arthritis. Curr Opin Rheumatol.

[87] Sun W, Meednu N, Rosenberg A, Rangel-Moreno J, Wang V, Glanzman J, Owen T, Zhou X, Zhang H, Boyce BF, Anolik JH, Xing L (2018). B cells inhibit bone formation in rheumatoid arthritis by suppressing osteoblast differentiation. Nat Commun.

[88] Choy EH, Panayi GS (2001). Cytokine pathways and joint inflammation in rheumatoid arthritis. N Engl J Med.

[89] O'Hayre M, Salanga CL, Handel TM, Allen SJ (2008). Chemokines and cancer: migration, intracellular signalling and intercellular communication in the microenvironment. Biochem J.

[90] Koch AE, Kunkel SL, Harlow LA, Johnson B, Evanoff HL, Haines GK, Burdick MD, Pope RM, Strieter RM (1992). Enhanced production of monocyte chemoattractant protein-1 in rheumatoid arthritis. J Clin Invest.

[91] Akahoshi T, Wada C, Endo H, Hirota K, Hosaka S, Takagishi K, Kondo H, Kashiwazaki S, Matsushima K (1993). Expression of monocyte chemotactic and activating factor in rheumatoid arthritis. Regulation of its production in synovial cells by interleukin-1 and tumor necrosis factor. Arthritis Rheum.

[92] Lau LF (2001). CCN1/CYR61: the very model of a modern matricellular protein. Cell Mol Life Sci.

[93] Chen CY, Fuh LJ, Huang CC, Hsu CJ, Su CM, Liu SC, Lin YM, Tang CH (2017). Enhancement of CCL2 expression and monocyte migration by CCN1 in osteoblasts through inhibiting miR-518a-5p: implication of rheumatoid arthritis therapy. Sci Rep.

[94] Peng M, Wang Y, Qiang L, Xu Y, Li C, Li T, Zhou X, Xiao M, Wang J (2018). Interleukin-35 Inhibits TNF-α-Induced osteoclastogenesis and promotes apoptosis *via* shifting the activation from TNF Receptor-Associated Death Domain (TRADD)-TRAF2 to TRADD-Fas-associated death domain by JAK1/STAT1. Front Immunol.

[95] Yuan N, Yu G, Liu D, Wang X, Zhao L (2019). An emerging role of interleukin-23 in rheumatoid arthritis. Immunopharmacol Immunotoxicol.

[96] Quinn JM, Sims NA, Saleh H, Mirosa D, Thompson K, Bouralexis S, Walker EC, Martin TJ, Gillespie MT (2008). IL-23 inhibits osteoclastogenesis indirectly through lymphocytes and is required for the maintenance of bone mass in mice. J Immunol.

[97] Kamiya S, Nakamura C, Fukawa T, Ono K, Ohwaki T, Yoshimoto T, Wada S (2007). Effects of IL-23 and IL-27 on osteoblasts and osteoclasts: inhibitory effects on osteoclast differentiation. J Bone Miner Metab.

[98] Ginting AR, Hidayat R, Sumariyono S, Koesnoe S (2020). Role of secreted frizzled-related protein 1 and tumor necrosis factor-α (TNF-α) in bone loss of patients with rheumatoid arthritis. Int J Rheumatol.

[99] Tang M, Lu L, Yu X (2021). Interleukin-17A interweaves the skeletal and immune systems. Front Immunol.

